# Polyphenols and Their Nanoformulations: Protective Effects against Human Diseases

**DOI:** 10.3390/life12101639

**Published:** 2022-10-19

**Authors:** Santosh Anand, Ramachandregowda Sowbhagya, Mohammad Azam Ansari, Mohammad A. Alzohairy, Mohammad N. Alomary, Asiyah I. Almalik, Wasim Ahmad, Takshashila Tripathi, Abozer Y. Elderdery

**Affiliations:** 1 Department of Biotechnology, School of Applied Sciences, REVA University, Bangalore 560064, Karnataka, India; 2 Department of Biotechnology and Genetics, Ramaiah College of Arts, Science and Commerce, Bangalore 560064, Karnataka, India; 3 Department of Epidemic Disease Research, Institute for Research and Medical Consultations (IRMC), Imam Abdulrahman Bin Faisal University, Dammam 31441, Saudi Arabia; 4 Department of Medical Laboratories, College of Applied Medical Sciences, Qassim University, Buraydah 52571, Saudi Arabia; 5 National Centre for Biotechnology, King Abdulaziz City for Science and Technology (KACST), Riyadh 11442, Saudi Arabia; 6 Maternity and Children Hospital, Buraydah 51452, Saudi Arabia; 7 Department of Pharmacy, Mohammed Al-Mana College for Medical Sciences, Dammam 34222, Saudi Arabia; 8 Department of Neuroscience, Physiology & Pharmacology, University College London, London WC1E 6BT, UK; 9 Department of Clinical Laboratory Sciences, College of Applied Medical Sciences, Jouf University, Sakaka 72388, Saudi Arabia

**Keywords:** polyphenols, bioavailability, nanoformulations, human health, nanonization, cancer, neurological diseases, cardiovascular diseases

## Abstract

Polyphenols are the secondary metabolites synthesized by the plants as a part of defense machinery. Owing to their antioxidant, anti-inflammatory, anticancerous, antineoplastic, and immunomodulatory effects, natural polyphenols have been used for a long time to prevent and treat a variety of diseases. As a result, these phytochemicals may be able to act as therapeutic agents in treating cancer and cardiovascular and neurological disorders. The limited bioavailability of polyphenolic molecules is one issue with their utilization. For the purpose of increasing the bioavailability of these chemicals, many formulation forms have been developed, with nanonization standing out among them. The present review outlines the biological potential of nanoformulated plant polyphenolic compounds. It also summarizes the employability of various polyphenols as nanoformulations for cancer and neurological and cardiovascular disease treatment. Nanoencapsulated polyphenols, singular or in combinations, effective both in vitro and in vivo, need more investigation.

## 1. Introduction

Polyphenols are plant secondary metabolites, with over 8000 polyphenolic compounds discovered to date, and have a variety of complicated structures [1]. The most common and abundant polyphenols are phenolic acids, phenolic alcohols, flavonoids, lignans, and stiblins, which are available in the plant kingdom. Polyphenols are thought to be significantly more effective than other antioxidants due to the diversity of their structures [2]. Polyphenols are a type of naturally occurring compound that has been shown to have the potential to be used as a therapeutic agent in the treatment of a diverse range of diseases. This is likely due to the fact that polyphenols serve as a form of defense for plants, that they are utilized in the food industry, and that they are excellent antioxidants. These bioactive substances are phytochemicals that help to protect people’s health from chronic degenerative diseases, which include cancer and cardiovascular and neurological disorders [3].

## 2. Classification of Polyphenols

Polyphenols are molecules with at least a single hydroxyl group present on an aromatic ring structure, and their backbone can range from a simple moiety to a complex polymer with a high molecular mass. The most widely accepted classification divides phenolics into two classes: flavonoids and non-flavonoid polyphenols [4]. The non-flavonoid polyphenols include phenolic acids, tannins, and stilbenes [5]. In this article, the health benefits of flavonoids, non-flavonoids, and their nanoparticles have been reviewed.

### 2.1. Flavonoids

**Figure 1:**
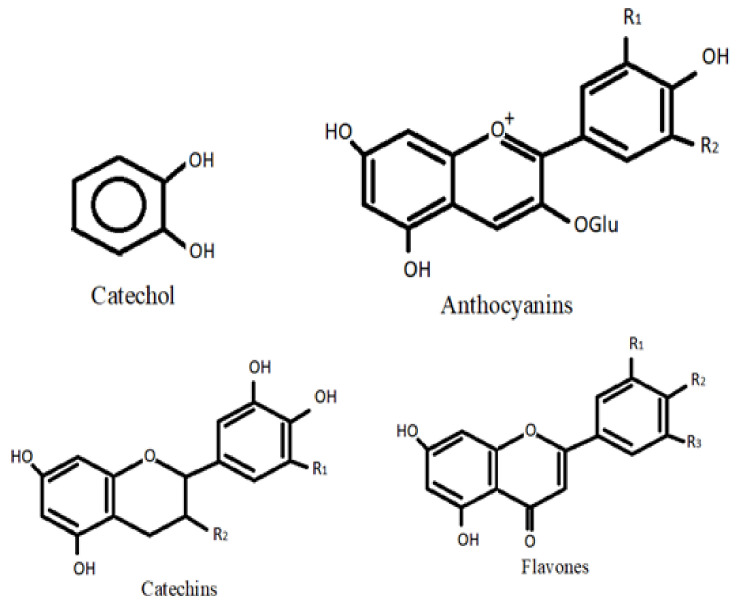
Flavonoids.

Flavonoids, which are low-molecular-mass phenolic moieties, are plant-derived molecules that can be found in various regions of plants existing in nature. Vegetables utilize flavonoids to enable them to survive and protect themselves against plaque. They are one of the major distinctive groups of compounds found in higher plants. In most angiosperm families, several flavonoids are easily recognized as floral pigments [6]. Flavonoids can be put into different subgroups based on where the B ring is linked to the C ring and how much the C ring is unsaturated and oxidized. Isoflavones are flavonoids in which the B ring is connected to the C ring at position 3. The compounds in which the B ring is attached at position 4 are referred to as neoflavonoids. In addition, when the B ring is connected to position 2, they can be further divided into several subclasses based on the structure of the C ring. Flavanols, flavones, flavanonols, flavanones, and flavanols, also called anthocyanins, catechins, and chalcones, are all parts of these subclasses [7] (Figure 1).

Choi et al. [8] have demonstrated the use of catechol to treat breast cancer, and Stat3 expression as a breast cancer stem cell (CSC) marker. According to them, catechol reduced the Stat3 expression and released IL-6, a CSC survival factor, inhibiting Stat3 signaling. Moon et al. [9] have reviewed the role of catechol in the enhancement of chemo and radio sensitivity by targeting AMPK/Hippo signaling in pancreatic cancer cells. Anthocyanins are usually found in fruits such as blueberries, raspberries, and black soybean, and also are found in vegetables, roots, legumes, cereals, and grains. These pigments dissolve in water. The blue and red colors of red grape and wine are because of the presence of phenolic moieties found in the skin of the berry and in the wine solution. Because they are easy to oxidize when under stress and because reactive oxygen species are present, these compounds are excellent antioxidants. They make fruits and vegetables better for human health, especially when it comes to degenerative and chronic diseases [10]. After 12 weeks of daily consumption of blueberry or Concord grape juice, the cognitive function in elderly persons improved [11,12]. Aged rats’ cognitive and motor abilities were enhanced by blueberries, bringing them up to par with young animals [13]. Catechins are naturally occurring polyphenolic flavanol compounds that can be present in a variety of culinary and medicinal plants, including tea, legumes, cocoa, berries, Fabaceae and Rubiaceae. Growing bodies of evidence have associated the consumption of foods high in catechins to the management of chronic human disorders such as inflammatory bowel disease (IBD) [14]. Baba et al. [15] have reported a positive impact on the working memory in Japanese adults between the ages of 50 to 60 years after 12 weeks of daily green tea catechins intake. Bernatoniene and Kopustinskiene [16] have reviewed the molecular characteristics of catechins, their antioxidant activity, and the mechanisms implicated in the prevention of oxidative-stress-related disorder. Flavones are a widely distributed flavonoid subgroup in plants that are produced by diverse pathways, depending on whether they possess a hydroxylated B-ring and C- or O-glycosylation [17]. Flavones have been substantially reported to be cancer-preventive. Diets rich in flavones reduce the risk of breast, skin, digestive, hematological, and prostate cancer. Flavones improve cardiovascular and neurological diseases besides cancer [18]. Reduced energy efficiency, decreased food intake, and weight loss have all been associated with a higher flavonoid intake. In order to prevent and treat obesity, flavonoids may provide a beneficial economic strategy with few, if any, adverse effects [19].

### 2.2. Phenolic Acids—Hydroxycinnamic and Hydroxybenzoic Acids

**Figure 2:**
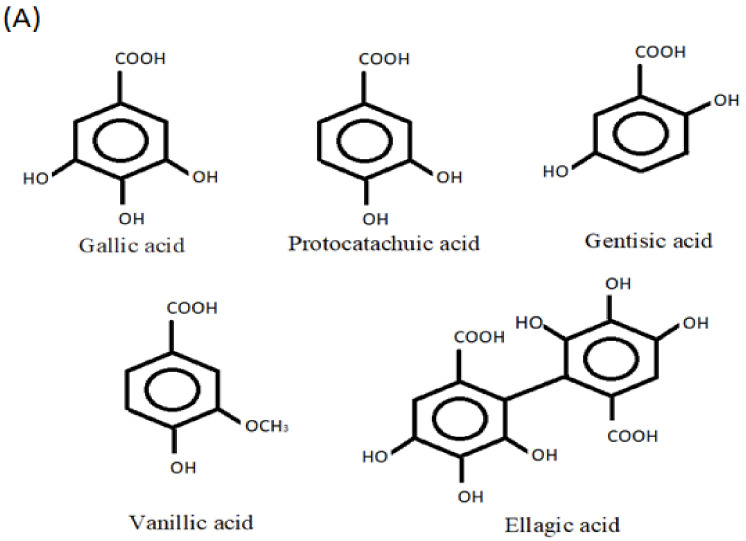

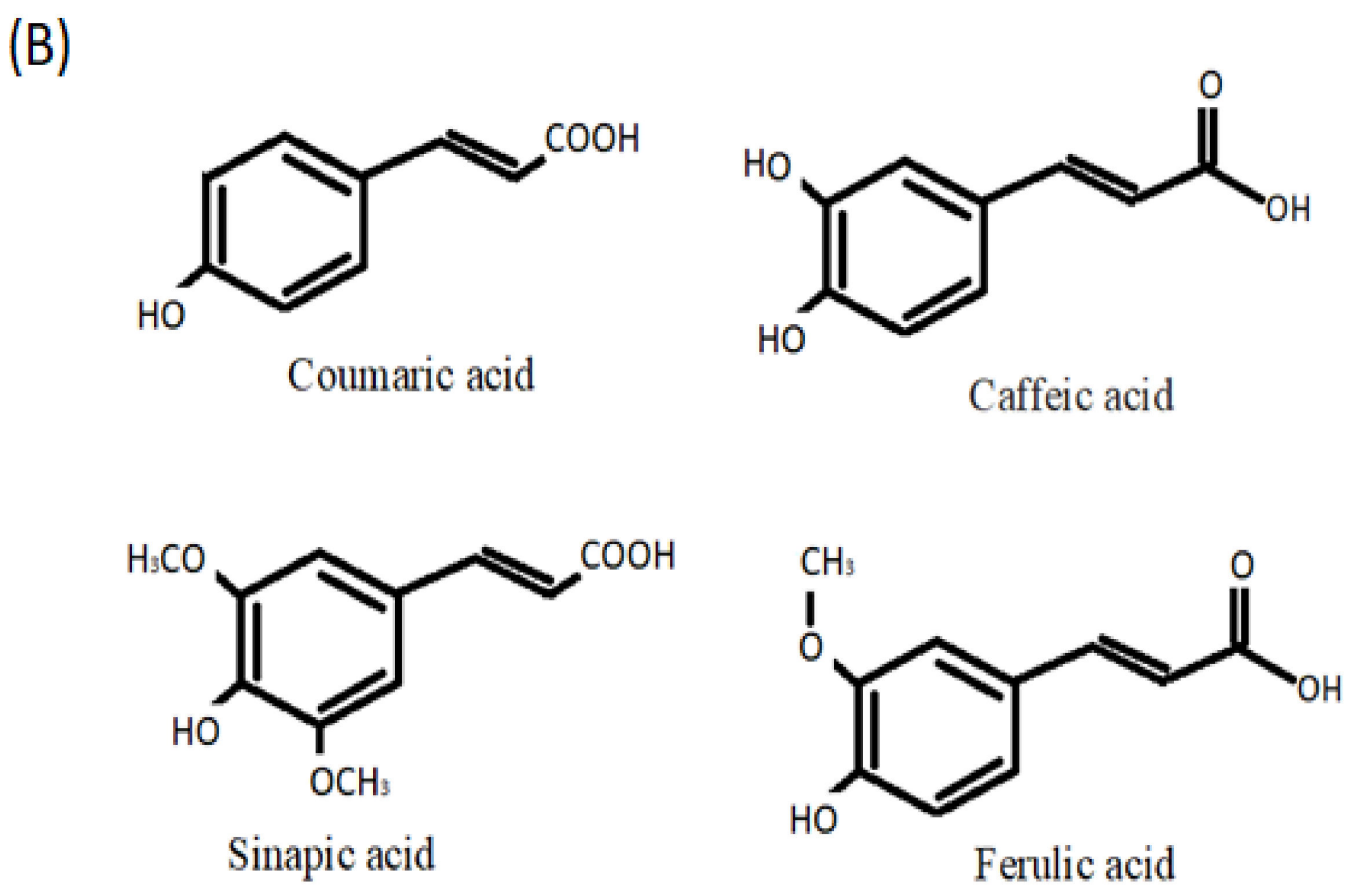
Phenolic acids: (**A**) hydroxycinnamic acid and (**B**) hydroxybenzoic acid.

The two types of phenolic acids are hydroxycinnamic and hydroxybenzoic acids (Figure 2A,B). Hydroxybenzoic acids in the human diet are uncommon, which is why they are not thought to play a function in human health. Cinnamic acid and benzoic acid derivatives have C1-C6 and C3-C6 backbones, respectively [20]. Phenolic acids can have neuroprotective and pro-cognitive effects through a variety of processes that vary depending on the natural substances that make up this diverse category [21]. By modulating intracellular calcium concentrations, chlorogenic acid has been shown to protect primary neurons against glutamate neurotoxicity [22] and has shown to have both neuroprotective and inflammatory effects in microglial cells infected with the herpes simplex virus [23].

### 2.3. Hydroxycinnamic Acids

Gallic acid is an effective antioxidant with neuroprotective and anti-inflammatory properties that has been shown to reduce cytokine levels in microglia cells and protect neurons from induced neurotoxicity by inhibiting NF-κB acetyltransferase [24]. Protocatechuic acid (PCA) is a form of naturally occurring phenolic acid that is extensively distributed in plants. PCA is a potent antioxidant with antibacterial, anticancer, antiasthma, antiulcer, anti-aging, antiathrogenic, antispasmodic, antitumoral, and neurological properties [25,26]. In experimental piglets, Hu et al. [27] found that dietary supplementation with PCA enhanced the expression of tight junction proteins such as claudin-1 and ZO-1 in the intestinal mucosa and reduced the serum concentration of thiobarbituric acid reactive substances (TBARS) when compared to control animals. Further, PCA supplementation has also been documented to reduce the levels of TNF and IL-2 in the blood.

Gentisic acid has numerous pharmacological activities, such as antioxidant, hepatoprotective, antimicrobial, anticarcinogenesis, analgesic, neuroprotective, and cardioprotective [28]. Gentisic acid increased the proliferation of keratinocytes. A Western blot analysis of proteins in the mitogen-activated protein (MAP) kinase signaling pathway revealed that ERK1/2 phosphorylation was augmented by gentisic acid supplementation. Thus, gentisic acid increases keratinocyte proliferation by phosphorylating EEK1/2 [29]. Vanillic acid has been shown to have antioxidant, anti-inflammatory, immunostimulatory, neuroprotective, hepatoprotective, cardioprotective, and antiapoptotic effects. According to reports, vanillic acid has the ability to reduce A1-42-induced cognitive impairment and oxidative stress, and hence aids in the treatment of Alzheimer’s disease [30]. In mice, vanillic acid reduces the severity of dextran sulfate sodium-induced ulcerative colitis, demonstrating its potential for controlling chronic intestinal inflammation [31]. Cikman et al. [32] have demonstrated that syringic acid lowered oxidative stress indicators and increased the antioxidant capacity in rats with L-arginine-induced acute pancreatic damage. Ellagic acid, an antioxidant active organic heterotetracyclic phenolic molecule found in grains and fruits, has been shown to have better accessibility to the brain and to increase the levels of antioxidant enzymes in aged rats [33].

### 2.4. Hydroxybenzoic Acids

Coumaric acid is a polyphenolic compound present in a wide range of plant-based diets, and studies have indicated that this phenolic molecule possesses neuroprotective properties [34]. An in vivo study has shown that coumaric acid supplementation reduces axonal degeneration in rat sciatic nerves and oxidative stress following ischemia/reperfusion [35]. Caffeic acid appears to have neuroprotective properties via reducing neuroinflammation and oxidative stress and, in particular, it was found to reduce sickness behavior and neuroinflammation in mice generated by lipopolysaccharides [36]. The phenethyl ester of caffeic acid has been demonstrated to be neuroprotective in rats exposed to ionizing radiation, reducing radiation-induced oxidative damage by improving SOD activity and lowering MDA levels in the brain [37]. Sinapic acid found in berries and cereals has been demonstrated to have neuroprotective properties in animal models of neurodegenerative diseases, including Alzheimer’s and Parkinson’s diseases [38,39]. Ferulic acid has shown its efficacy as a neuroprotective phenolic acid by increasing the levels of serotonin and norepinephrine in the mouse frontal cortex and hippocampus, but not dopamine levels, which are important in the pathophysiology associated with mood disorders [40].

### 2.5. Tannins

**Figure 3:**
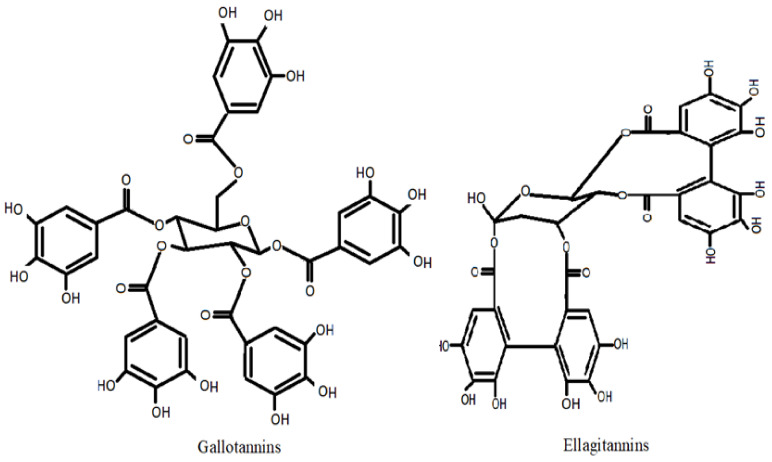
Structures of tannins.

Tannins are polyphenols with 500 to 30,000 Da MW and are divided into pyrogallol tannins or hydrolysable tannins and proanthocyanidins or condensed tannins. Hydrolysable tannins are constituted of phenolic acid esters and a polyol, generally glucose, which are gallotannins and ellagitannins [41]. A tannin, a water-soluble polyphenol, is double-edged. According to studies, the amount of tannins that causes a disorder in one animal does not affect another of the same species [42]. Tannins prevent lipid peroxidation and scavenge pro-oxidant free radicals, and most tannin activities, including free radical-scavenging, depend on structure and polymerization [43,44]. Tannin-enriched fractions isolated from *Myracrodruou urundeuva* on rat primary mesencephalic cells decreased 6-hydroxydopamine-induced neuronal cell toxicity. It reversed 6-hydroxydopamine-induced lipid peroxidation, indicated by the down-regulation of thiobarbituric acid reactive substances [45]. Senobari et al. [46] have reviewed the anticancer, chemoprotective, and anti-inflammatory properties of ellagitannins and their derivatives. Various factors could modulate pro-inflammatory mediators and growth factors, such as IL-6, TNF, TGF, IL-1, IFN, and ellagitannins, that target cancer stem cells and disrupt stem cell signaling. Figure 3 represents the structures of gallotannins and ellagitannins.

### 2.6. Stilbenes

**Figure 4:**
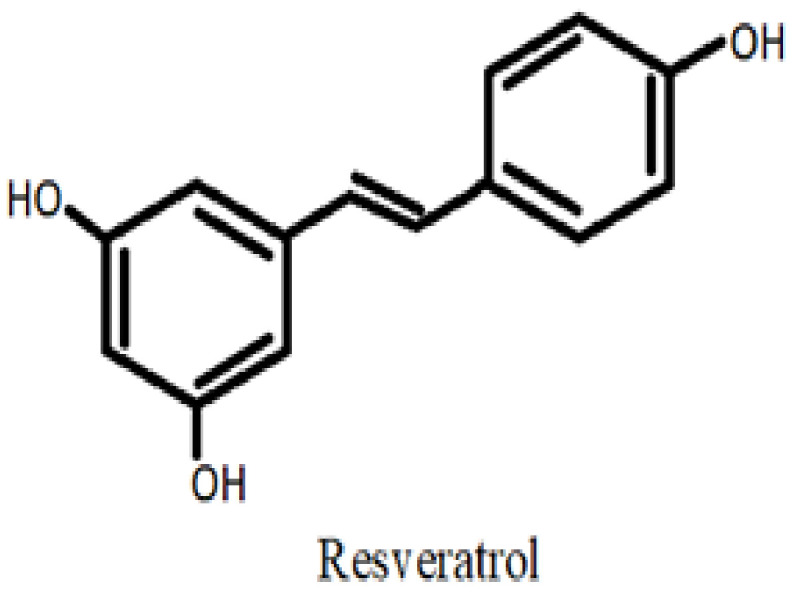
Stilbenes.

Although stilbenes are not considered to be flavonoids, their chemical makeup and biological activities are quite similar to those of flavonoids. Different plants naturally produce stilbenes, which are produced in reaction to pathogen infection (phytoalexins), exposure to ultraviolet radiation, and involvement in bacterial root nodulation and coloring [47]. A methylene bridge connects the two aromatic rings (rings A and B) that make up the chemical structure of stilbenes. Trans-resveratrol, a member of the stilbene family that has been hydroxylated at the 3, 5, and 4 positions, is one of the most well-known molecules [48] (Figure 4). Recent studies of Ma et al. [49] have revealed the neuroprotective activities of resveratrol in rat models of AD and diabetes. Resveratrol significantly increased the expression of Sirt1 and stopped memory loss, high levels of malondialdehyde, acetylcholinesterase, interleukin-6, and interleukin-1b, and lowered levels of superoxide dismutase (SOD), choline acetyltransferase (ChAT), and glutathione. In another study, resveratrol reduced the inflammation and oxidative stress in the kidneys of diabetic rats, and the treatment upregulated the gene and protein expression profile of NF-κB in them.

**Figure 5:**
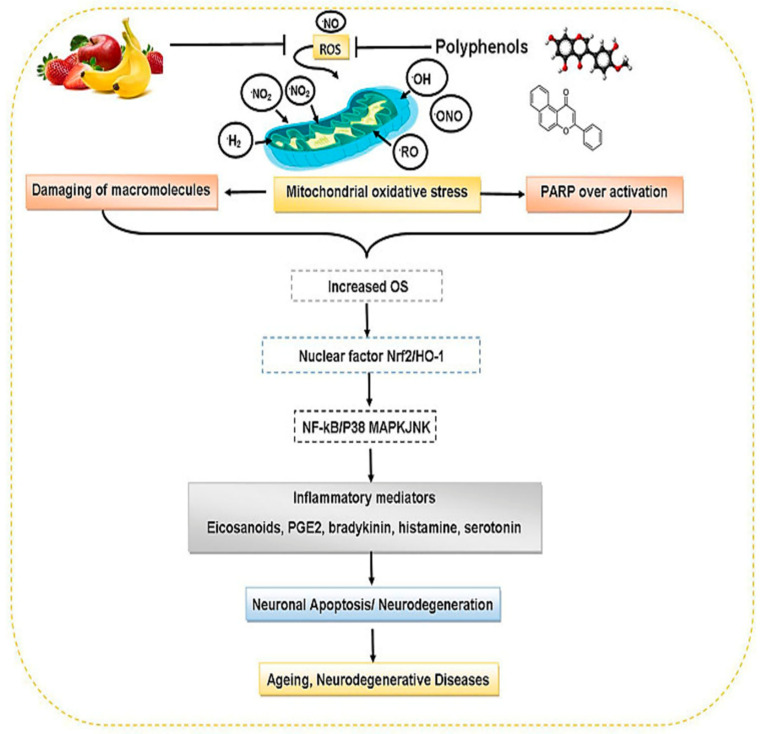
Protective roles of dietary polyphenols against aging and neurodegenerative disorders (adapted from reference [65]).

Exploring the therapeutic effects of natural polyphenols in the prevention and treatment of cardiovascular and neurological illnesses, particularly cancer, has received substantial attention in scientific research in recent years [50]. Plant sources have reported varied concentrations of these polyphenolic compounds (Table 1). Figure 5 demonstrates the preventive roles of dietary polyphenols against aging and neurodegenerative diseases.

**Figure 6:**
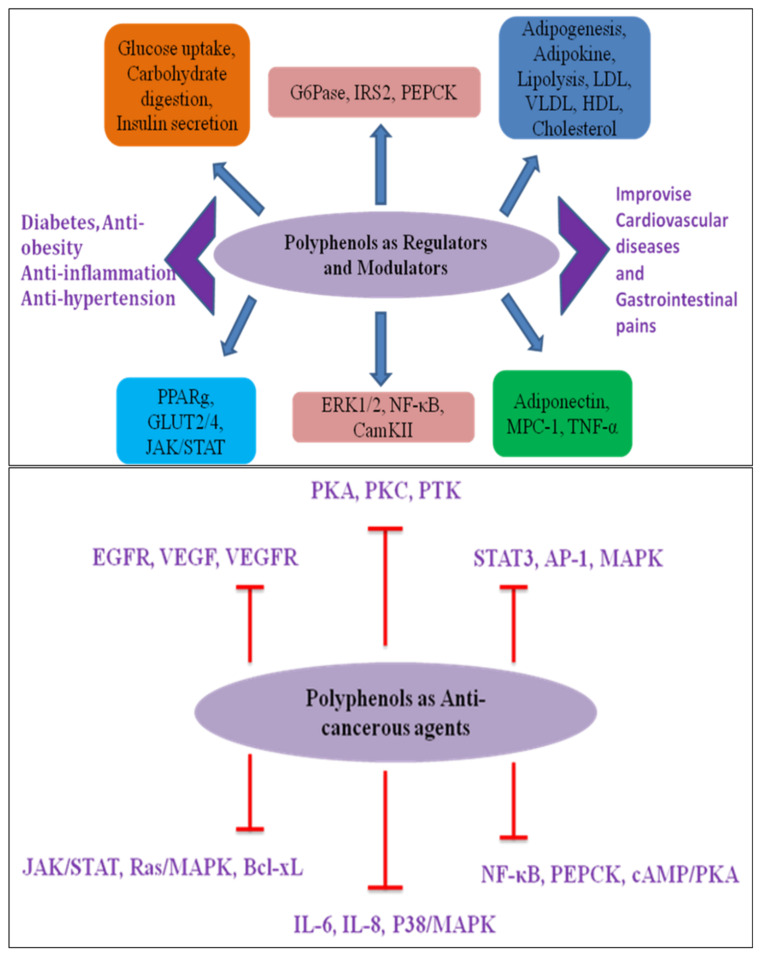
Possible molecular targets of polyphenols orchestrating human health benefits as regulators, modulators, and anticancerous agents.

Preclinical and clinical research suggest that an increased intake of polyphenol-rich diets protect against neurodegenerative disorders, cardiovascular diseases, cancer, diabetes, inflammatory disorders, and infectious illnesses. It has been suggested that the antioxidant activity of dietary polyphenols plays a crucial role in preventing oxidative-stress-induced diseases in humans [65]. The possible molecular targets of polyphenols orchestrating human health benefits as regulators, modulators, and anticancerous agents are represented in Figure 6.

## 3. Nanoformulations of Polyphenols

Dietary polyphenols have been studied for their medicinal potential, either as singular or in combination. These polyphenols have shown potential therapeutic properties in vitro and in vivo. Despite their beneficial consequences, a number of characteristics, including a low solubility, poor permeability, instability, and a rapid release of the compounds, are some of the limitations to the study of their clinical applications [66]. The other factors, such as an inefficient systemic administration, stability, and low bioavailability, also limit their clinical benefits as therapeutics. The nanoformulation of polyphenols is one potential alternative to this challenge, and has produced some intriguing findings [67]. Polyphenol nanoencapsulation could extend circulation, improve localization, boost efficacy, and lower the risk of multidrug resistance. There is evidence that nanocarriers are superior materials for encapsulating phenolic chemicals and increasing their bioavailability [68]. A wide range of organic, inorganic, or organic/inorganic hybrid nanomaterials have been fabricated that have unique physical and chemical properties. Nanomaterials, which range in size from 1 to 100 nm, have comparatively extended surface areas, created for a variety of biomedical applications, such as disease therapy, imaging, and sensing. However, the acute toxicity or long-term adverse effects of these engineered nanomaterials raise major health concerns. As a result, it is vital to develop new types of nanomaterials with a high performance and biocompatibility [69].

### 3.1. Encapsulation of Polyphenols and Their Controlled Release

Encapsulation is a common way to make a layer or membrane of one material on the outside of another material. This is carried out to protect or keep bioactive, volatile, or easily degradable compounds from biochemical and thermal breakdown. In encapsulation processing, nanocapsules and microcapsules are the most useful and sought-after sizes. Even though nano scale and micro scale mean 1–1000 nm and 1–1000 m, respectively, the size of a nano-encapsulated capsule can be anywhere from 1 nm to a few hundred nm in diameter, and the size of a micro-encapsulated capsule can be anywhere from 1 m to a few hundred meters in diameter. In addition, particles with sizes between nano and micro encapsulation are called submicron particles, and particles with sizes above microencapsulation are called macro-particles. Microencapsulation is the most common and widely used method. Nanoencapsulations have become more popular recently because they have a unique feature that makes them more stable and gives them more control over how materials inside them are released [70]. Nanoencapsulation is a good way to make polyphenols more soluble, slow down their breakdown, lower their toxicity, and control their active absorption and biological response. Nanoencapsulation is a broad term for a number of different methods that are based on chemical, physical, and physiochemical principles. Chemical nanoencapsulation (such as interfacial and in situ polymerization) requires the polymerization of monomers at the interface of two immiscible substances by adding a cross-linker in the external phase. Physical processes, such as the air-suspension method, pan coating, spray drying, spray congealing, micro-orifice system, and so on, involve the interaction of the vector material with the molecules to be encapsulated when both are aerosolized or atomized. Lastly, particle size reduction techniques such as coacervation, phase separation, complex emulsion, meltable dispersion, and nanoprecipitation can be used to make stable nanometer-sized drug nanosuspensions or nanoparticles. Physiochemical methods are interesting ways to improve the pharmacological action because they can be used in a variety of ways and can increase loading capacities, persistence at the target sites, and permeation and retention effects. Several studies have been conducted recently on the nanoparticle-mediated delivery of polyphenols. These studies use biodegradable and biocompatible polymers that can deliver polyphenols [71,72].

Nanospheres (NS) are another sort of nanosized vectors, with hydrophobic chains inside and hydrophilic parts outside. These nano drug delivery systems contain homogeneous solid matrices in which polymer chains are “frozen”. Nanospheres can be fine-tuned by using different shell materials, such as poly-lactic acid (PLA), poly-glycolic acid (PGA), poly-lactic-co-glycolic acid (PLGA), poly-caprolactone (PCL), chitosan (CS), polyethylene glycol (PEG), and Eudragit (anionic copolymers based on methacrylic acid and methyl methacrylate). Biocompatible and biodegradable polymers are used to make NS. Drugs are dissolved, entrapped, encapsulated, chemically bonded, or adsorbed to the polymer matrix. Nanocapsules (NCs) have a similar composition but with a core–shell construction in which the medicine is encased in a polymer membrane. Nanocapsules can carry active ingredients on their surfaces or in their layers. Active chemicals are liquid or solid [73]. Cyclodextrins (CDs) are generated when bacteria breakdown cellulose. They are cyclic oligosaccharides with a lipophilic center chamber and hydrophilic outside surface. These compounds are water-insoluble. Water-soluble cyclodextrin derivatives such as hydroxypropyl βCD and γCD, randomly methylated -cyclodextrin, and sulfobutylether -cyclodextrin sodium salt produce effective nano-drug delivery systems. CDs create compounds with molecules through van der Waals, hydrophobic, or hydrogen bonding. This complexation is increased by an opposing charge and low temperature [74].

Micelles (MCs) are 5 to 100 nm colloidal dispersions that develop spontaneously from amphiphilic substances at particular concentrations and temperatures. MC formation is driven by a decrease in free energy due to the removal of hydrophobic pieces from the continuous phase and the re-establishment of hydrogen bonds in water. Moreover, additional energy occurs from the development of Van der Waals bonds between hydrophobic blocks in the center of the produced micelles. Hydrophobic pieces of amphiphilic molecules create micelle cores. MCs are stable, biocompatible, and solubilize poorly soluble drugs. Pluronic, poly (ethylene glycol), poly (D,L-lactide-co-glycolide), and polycaprolactone (PCL) are amphiphilic agents used to create MCs [75]. Solid lipid nanoparticles (SLNs) are vectors made of high-melting-point lipids and aqueous surfactants. Fatty acids, acylglycerols, and waxes are core lipids; phospholipids, sphingomyelins, bile salts, and sterols are stabilizers. SLNs have a high biocompatibility, high bioavailability, physical stability, protection of incorporated labile drugs from degradation, excellent tolerability, prevention of multiple routes of administration problems, avoidance of organic solvents during preparation, and absence of large-scale production and sterilization problems. Particle growth, unpredictable gelation tendency, unclear drug distribution inside the vector’s lipid matrix, and unanticipated polymorphic transition kinetics are disadvantages of SLNs [74]. Liposomes (LS) are drug delivery systems that develop spontaneously by hydrating lipid powder in aqueous media. Liposomes have particle sizes ranging from 30 nm to several micrometers and consist of one or more lamellae (phospholipidic bilayer membranes) surrounding aqueous units. Polar lipids self-assemble into colloidal particles based on the molecule shape, temperature, and dispersion conditions [76]. Lipid nanoparticles can carry hydrophilic and lipophilic compounds. Biocompatibility, biodegradability, scalability, and controlled/extended release are lipid nanoparticles’ key benefits over colloidal and polymeric nanoparticles [77].

### 3.2. Characterization of Nanoformulated Polyphenols

There are instances when just one method of characterization can be used to fully understand a property, and other times when multiple methods are used in tandem. There are microscopy-based techniques (such as transmission electron microscopy (TEM), high-resolution transmission electron microscopy (HRTEM), and atomic force microscopy (AFM)) that provide details about the nanomaterials’ dimensions, shape, and crystal structure. Some methods, such as magnetic ones, are tailored to specific classes of materials. SQUID, VSM, FMR, and XMCD are all examples of such methods. In addition to these methods, many others can be used to learn more about the nanoparticles’ structure, elemental composition, optical properties, and other general and specialized physical characteristics. Methods such as X-ray imaging, spectroscopy, and scattering are also the other instruments used to characterize them [78].

Transmission electron microscopy is one of the most crucial methods for characterizing nanoformulated polyphenols. Micrographs of nanoscale materials with great lateral spatial resolution can be obtained using TEM by focusing an electron beam on a thin (usually less than 200 nm) sample. By minimizing distortion in the image with aberration correctors, modern electron microscopes may provide high-resolution images with atomic resolution, down to a resolution of 0.05 to 0.1 nm. By spatially restricting and concentrating the impinging beam and detecting the resulting electron diffraction pattern, TEM also allows for the investigation of the crystalline structure of specific microscopic sections of crystalline materials [79]. A scanning electron microscope can make a picture of the surface of a sample by picking up the secondary electrons that are emitted when an electron beam hits the sample. In SEM, samples are imaged with beams that have less energy than those used for TEM characterization. This means that the beam can only go so far into the sample and is only sensitive to the surface. However, this simple interaction also means that SEM characterization can be used to look at the shape of “thick” (>100 nm) samples, which TEM cannot do. The low electron energies used in SEM analysis usually limit the resolution to >2–3 nm. However, compared to TEM, this makes it much less likely for the beam to damage the sample. In addition, SEM is much easier to use, allows measurements to be carried out faster, and costs less to buy and keep up than TEM [78]. Scanning probe microscopy methods, such as atomic force microscopy, can be used to probe and observe the surface (and several other force-related characteristics) of nanometer- or even atomic-sized objects. When a laser beam is reflected off the sharp tip of a cantilever and onto a photodiode array, the forces experienced by the cantilever as a result of its interaction with the sample can be recorded. This may involve a change in the amplitude, frequency, or phase of an oscillating cantilever, or it may be a vertical or lateral deflection of the cantilever, depending on the measurement mode [80].

### 3.3. Cancer Therapy

Cancer is described as an aberrant cell proliferation that can result in malignancies that are life-threatening and incur significant financial burdens on both patients and the healthcare system. Natural polyphenols have been employed for a long time to prevent and treat a variety of diseases. As a result, these phytochemicals may have the ability to act as chemotherapeutic and chemopreventive agents in various cancers due to their anticancerous properties [81]. Study in human cervical cancer KB-3-1 and KB-V1 cells, curcumin-loaded PLGA nanoparticles coupled with anti-P-glycoprotein, exhibited cytotoxic properties, leading to an augmented curcumin solubility and cellular uptake in addition to lower cell survival [82]. Yu et al. [83] have created ferric-coordinated polyphenol nanoparticles to activate PTT-aided ferrous therapy for the treatment of cancer. Pirzadeh-Naeeni et al. [84] have reported the anticancerous properties of ellagic acid loaded with schizophyllan and chitin nanoparticles in MCF-7 breast cancer cells. A cell viability assessment revealed substantial antiproliferative effects on MCF-7 cells, which were enhanced at higher dosages. In a recent work, Ghayour et al. [85] reported the nanocapsulation of quercetin and curcumin in casein-based delivery systems, which were tested against MCF-7 cell lines. In this case, the capsulated polyphenols reduced the progression of tumor cells when compared to the non-capsulated ones. The *Oringa majorana* L. leaf extract used in the green technique to create cerium nanoparticles showed all of the traits of a functional nanoparticle and had the ability to raise the gene expression levels of the principal antioxidant-related enzymes, CAT and SOD. Compared to normal cells, the cerium oxide nanoparticles (CeO-NP) demonstrated more cytotoxicity against breast cancer. In light of this, CeO-NP appeared to be a viable therapeutic agent for the treatment of breast cancer cells while shielding healthy cells from oxidative stress and inflammation brought on by free radicals [86].

### 3.4. Neuroprotective Effects

Oxidative stress is a condition where the scavenging antioxidant system is overpowered by high oxygen levels and oxygen-derived free radicals. These include substances such as peroxyl (ROO), hydroxyl (OH), and hydrogen peroxide (H_2_O_2_) radicals, as well as superoxide anions (O_2_). The Brain consumes 20% of the total basal oxygen and experiences one of the greatest extents of oxidative stress of any organ in the body since it is the most highly oxidative organ [87]. Superoxide dismutase, glutathione/glutathione peroxidase, catalase, and single-molecule antioxidants such as vitamins E and C are just a few of the endogenous processes that detoxify oxidative damage. However, in many disease conditions, free radical generation outpaces natural defensive mechanisms, leading to greater levels of oxidative stress. A significant amount of oxidative stress in the brain is linked to the majority of neurodegenerative ailments, including Parkinson’s, Alzheimer’s, Huntington’s, multiple sclerosis, traumatic brain injury, ischemia, and aging itself [88]. A growing body of evidence has revealed the neuroprotective effects of polyphenols in attenuating oxidative stress in the brain [33,89]. Due to its high regenerating antioxidant characteristics, cerium oxide nanoparticles are widely used in the materials industry and are currently being considered for use in biomedicine. Cerium oxide nanoparticles are being researched for their potential to treat a number of neurodegenerative diseases and have already demonstrated promising levels of neuroprotection [90]. Chen et al. [91] have investigated the neuroprotective effects of CeO2@SiO2-PEG nanoparticles (CSP-NPs) for the delivery of proanthocyanidin and curcumin. Curcumin (Cur) and proanthocyanidin (PAC) were loaded onto CeO2@SiO2-PEG nanoparticles to create Cur-NPs and PAC-NPs, two hydrophilic and hydrophobic compounds. In PC-12 cells, Cur-NPs and PAC-NPs exhibited a potent acetylcholinesterase (AchE) inhibitory property, as well as advantageous neuroprotective properties against A1-42-mediated toxicity. Various curcumin-loaded nanoparticle systems, such as poly (-caprolactone) (PCL), poly (lactide-co-glycolide) (PLGA), or methoxy poly (ethylene glycol) poly (-caprolactone (MPEG-PCL), have been described in numerous investigations. These curcumin-loaded nanoparticle systems boost curcumin levels in the bloodstream and enhance its chemical stability, inhibiting enzymatic and pH breakdown and thereby exhibiting its neuroprotective properties [92]. PEGylated PLGA nanoparticles that are loaded with two drugs (epigallocatechin-3- gallate and acetyl acid) have the potential to be created as a secure and effective therapeutic alternative for the treatment of Alzheimer’s disease. When mice were given epigallocatechin-3- gallate/ascorbic acid NPs orally, epigallocatechin-3- gallate accumulated in all of the body’s major organs, including the brain. It has been demonstrated that this formulation may boost the drug’s persistence in the bloodstream and brain tissue [93]. Ravikiran et al. [94] have reported a significant reduction in the cytotoxicity of H_2_O_2_ in PC12 cells by the 4-hydroxyisophthalic acid (4-HIA) encapsulated PLGA-NPs compared to non-encapsulated 4-HIA.

### 3.5. Cardioprotection

It has been hypothesized that oxidative stress plays a vital role in the aetiology of heart disease. A reduced antioxidant reserve and increased oxidative stress are linked to both acute and chronic heart failure [95]. Polyphenols have the ability to protect against oxidative-related illnesses, including cardiovascular disease: notably, ischemic heart disease and stroke caused by atherosclerosis. Several studies have reported the cardioprotective effects of polyphenols against oxidative-related illness [96,97]. To modulate the drug discharge profile, Wang et al. [98] placed a bioactive polymer (PLGA layers) onto superparamagnetic SiN.SiN@QC-PLGA nanobiocomposite and enhanced the practical resemblance to the local myocardial by enabling the cell recruitment, expansion, attachment, and articulation of cardiac proteins, which can be used to prevent atherosclerosis and other cardiovascular illnesses. Hesari et al. [99] have reviewed the effectiveness of recently developed nanoformulated natural remedies against a variety of cardiovascular disorders, including hypertension, atherosclerosis, thrombosis, and myocardial infarction. Carlson et al. [100] investigated the cardioprotective potential of curcumin nanoformulation in cardiomyocytes. In rat embryonic cardiomyocytes (H9C2) treated with doxorubicin hydrochloride, the experimental method revealed that Cur-Res-mP127 (co-loaded curcumin and resveratrol at a molar ratio of 5:1 in Pluronic^®^ F127 micelles) decreased apoptosis and ROS, indicating cardioprotection. Table 2 represents the disorder/disease treated with polyphenols loaded with nanoparticles employing conjugation and encapsulation strategies.

### 3.6. Bioavailability of Nanoformulated Polyphenols

**Figure 7:**
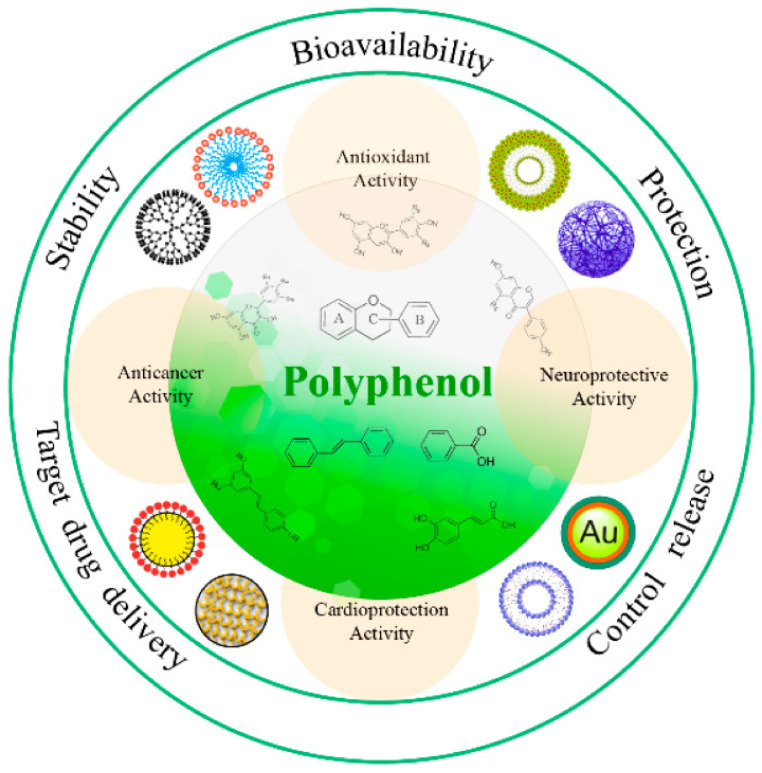
Schematic representation of nanoformulations to enhance the bioavailability and physiological functions of polyphenols (Adapted from reference [68]).

There are different routes of administrating nanoformulated polyphenols, which include topical, oral, intravenous, intraocular, pulmonary, and intracranial [77]. To improve the bioavailability and stability of natural polyphenols and, consequently, their therapeutic efficiency, a variety of nano-formulations, including lipid nanoparticles, micelles, liposomes, and polymeric nanoparticles, have been developed. The capacity of nanoparticles to permeate the blood–brain barrier plays a key role in neuroprotection, in addition to extending the half-life [101]. An improved bioavailability, targeted/controlled release, and active ingredients protection are all possible by optimizing the nanoparticle size, which ranges between 1–100 nm. Numerous nanoparticles, including phospholipid complexes, protein-based nanoparticles, liposomes, niosomes, emulsions, micelles, and metal nanoparticles, have been created in recent years for the delivery of phenolic compounds [68] (Figure 7). The three most well-known bioactive dietary polyphenols—epigallocatechin-3-gallate, resveratrol, and curcumin—have a low bioavailability. Reassembled proteins, protein–polysaccharide conjugates (complexes), crosslinked polysaccharides, and emulsified lipids have all been used to create food macromolecule-based nanoparticles utilizing safe methods that can be used in food. The human digestive tract is the region where food-grade macromolecule nanoparticles have their greatest impact on increasing the dietary polyphenols’ bioavailability. This is achieved by making the polyphenols more soluble, preventing their degradation in the intestinal environment, increasing their permeability in the small intestine, and even raising the concentrations in the bloodstream [102]. In addition, the doses utilized in many in vitro experiments are nearly impossible to reach through dietary polyphenol consumption. Numerous studies have been devised to evaluate the size, stability, kinetics, and chemical properties of various microencapsulations, the majority of which have been conducted in vitro. However, a few studies have examined the impacts in in vivo models in order to assess the eventual impacts on the organism and various health issues [103]. Applying the nanotechnology to study the bioavailability in clinical trials is necessary to increase the knowledge related to the effects of nanoformulated polyphenols in humans.

## 4. Conclusions

Phenolic compounds, which are among the most abundant plant active substances, serve a variety of biological roles that are advantageous to human health. Moreover, the use of these molecules in food and medicine is severely constrained, owing to their poor stability, poor solubility, and low bioavailability. These restrictions can be circumvented by encapsulation in nanoparticles, which can also target and control their release. In order to enhance the pharmacokinetics and bioavailability of polyphenols, nanotechnology offers the perfect carrier system. Although nanoparticles are almost ideal as carriers, their side effects and toxicity must still be taken into account and reduced prior to clinical manifestation. Understanding the hazardous side effects pertaining to the nanoparticles accumulation in the physiological system is crucial because polyphenols are natural substances that must be taken for an extended time period for the treatment and prevention of diseases. This is especially true if the nanoparticles possess a lower encapsulation rate. Therefore, in order to encourage the development and implementation of novel efficacious nanoparticles advantageous to human health, standardized in vitro and in vivo models must be established, and in vivo safety testing methods need to be validated.

## Figures and Tables

**Table 1:** Concentrations of polyphenolic compounds in some plant sources.

Polyphenols	Examples	Source	Concentration (g/Kg)	References
Phenolic acid—Hydroxycinnamic acid	Gallic acid	Blackcurrant Primrose	30–62 mg/kg dry weight 15,000 mg/kg	[51] [52]
	Protocatechuic acid	Acai fruit	630 mg/kg	[53]
	Gentisic acid	Citrus fruit (*Citrus paradisi*)	30,000 mg/kg	[54]
	Vanillic acid	Acai fruit	1616 mg/kg	[53]
	Ellagic acid	Pomegranate Raspberry	700 mg/kg dry weight (arils) 38,700 mg/kg dry weight (mesocarp) 1500 mg/kg dry weight 2637–3309 mg/kg fresh weight	[55] [56] [57]
Phenolic acid—Hydroxybenzoic acid	Coumaric acid	Corn Barley	242 mg/kg 75 mg/kg	[58]
	Caffeic acid	Cabbage (SA)	42.5 mg/kg	[59]
	Sinapic acid	Lemon Strawberry Cranberries	72.1 mg/kg 450.1 mg/kg 210 mg/kg	[60]
	Ferulic acid	Acai fruit	101 mg/kg	[53]
Flavonoids	Anthocyanins	Plum peel Blueberry	604.5 mg/kg 223.8 mg/kg	[61]
	Catechin	Acai fruit	66.7 mg/kg	[53]
	Flavones	Fenugreek seed (Apigenin) (Luteolin)	7310 mg/kg 5120 mg/kg	[62]
Tannins	Ellagitannins	Tea	0.15 to 4.46 mg ellagic acid equivalent/g tea	[63]
Stilbenes	Resveratrol	*Morus alba* (fruit) *Rumex japonicas* (root)	7.95 × 10^−3^ 8.4 × 10^−3^	[64]

**Table 2:** An overview of polyphenol-loaded nanoparticles and the disorder/disease treated.

Disorder/Disease	Nanocarrier	Loading	Polyphenol/s	Cell Line/s	Result	References
Cancer	PLGA	Conjugation	Curcumin	KB-V1 and KB-3-1 cells	Cur-NPs had a considerably higher level of specific binding to KB-V1 cells than it did to KB-3-1 cells. In comparison to KB-3-1 cells, the cellular absorption of Cur-NPs-APgp was greater in KB-V1 cells.	[82]
	Schizophyllan and chitin nanoparticles	Encapsulation	Ellagic acid	MCF-7 cells	EA/SPG-NP and EA/Ch-NP substantially reduced the proliferation of breast cancer cell lines at IC50 values of 60 and 115 g/mL, respectively.	[84]
	Nanocapsules	Encapsulation	Curcumin and quercetin	MCF-7 cells	The cytotoxicity of bioactive compounds that were encapsulated was shown to be greater than that of free forms.	[85]
Neurological	CeO2@SiO2-PEG nanoparticles (CSP-NPs)	Encapsulation	Proanthocyanidins and curcumin	PC-12 cells	CSP-NPs effectively delivered proanthocyadinins and curcumin to exhibit potent neuroprotective effect against A1-42-mediated toxicity and recovered cell viability from 57.5% to 81.0% at 25 g/mL	[91]
	PLGA-NPs	Encapsulation	4-hydroxyisophthalic acid	PC-12 cells	4-HIA and 4-HIA encapsulated PLGA, dose-dependently reduced H_2_O_2_-induced toxicity, and restored cell viability to 84% and 94%, respectively.	[94]
Cardiovascular	Pluronic^®^ F127	Encapsulation	Curcumin and resveratrol	H9c2 cells	Combination of resveratrol and curcumin as free drugs or as micelles with doxorubicin hydrochloride inhibited apoptosis and sequestered ROS in H9C2 cells.	[100]

## Data Availability

It can be found on request.
